# Cattle Mammary Bioreactor Generated by a Novel Procedure of Transgenic Cloning for Large-Scale Production of Functional Human Lactoferrin

**DOI:** 10.1371/journal.pone.0003453

**Published:** 2008-10-20

**Authors:** Penghua Yang, Jianwu Wang, Guochun Gong, Xiuzhu Sun, Ran Zhang, Zhuo Du, Ying Liu, Rong Li, Fangrong Ding, Bo Tang, Yunping Dai, Ning Li

**Affiliations:** 1 State Key Laboratory for Agrobiotechnology, China Agricultural University, Beijing, People's Republic of China; 2 Beijing Genprotein Biotechnology Company, Beijing, People's Republic of China; Cairo University, Egypt

## Abstract

Large-scale production of biopharmaceuticals by current bioreactor techniques is limited by low transgenic efficiency and low expression of foreign proteins. In general, a bacterial artificial chromosome (BAC) harboring most regulatory elements is capable of overcoming the limitations, but transferring BAC into donor cells is difficult. We describe here the use of cattle mammary bioreactor to produce functional recombinant human lactoferrin (rhLF) by a novel procedure of transgenic cloning, which employs microinjection to generate transgenic somatic cells as donor cells. Bovine fibroblast cells were co-microinjected for the first time with a 150-kb BAC carrying the human lactoferrin gene and a marker gene. The resulting transfection efficiency of up to 15.79×10^−2^ percent was notably higher than that of electroporation and lipofection. Following somatic cell nuclear transfer, we obtained two transgenic cows that secreted rhLF at high levels, 2.5 g/l and 3.4 g/l, respectively. The rhLF had a similar pattern of glycosylation and proteolytic susceptibility as the natural human counterpart. Biochemical analysis revealed that the iron-binding and releasing properties of rhLF were identical to that of native hLF. Importantly, an antibacterial experiment further demonstrated that rhLF was functional. Our results indicate that co-microinjection with a BAC and a marker gene into donor cells for somatic cell cloning indeed improves transgenic efficiency. Moreover, the cattle mammary bioreactors generated with this novel procedure produce functional rhLF on an industrial scale.

## Introduction

Human lactoferrin (hLF) is a multifunctional glycoprotein of 80 kDa secreted in many tissue fluids including tears, saliva, semen, vaginal secretion, milk, and plasma[1]. Both *in vitro* and *in vivo* evidence indicate that hLF is involved in iron absorption in the intestinal tract [2] as well as in broad-spectrum primary defense against bacteria [3], fungi [4], protozoa [5] and viruses [6]. In addition, several studies also suggest that hLF modulates the inflammatory response [7], regulates gene expression [8], and promotes bone growth [9]. These bioactivities suggest that hLF may have important therapeutic applications, such as in prophylaxis treatment, nutritional supplementation, and food and/or medicine preservation. Therefore, market demand for hLF is primed to expand dramatically. A number of attempts have been made to produce recombinant human lactoferrin (rhLF) using prokaryotic and eukaryotic expression systems [10]–[16]. However, problems such as low protein expression level, lack of accurate post-translational modifications as well as complex purification procedures have made current approaches unsuitable for large-scale production. Recently, transgenic mice expressing rhLF were successfully established by Platenburg's group, which paved the way for harvesting rhLF by means of a mammary bioreactor [17], [18]. As such, a cattle mammary bioreactor would be an excellent system for large-scale production of rhLF because of its established faithful incorporation of post-translational modifications and efficiency for purification of heterologous proteins.

To date, more than ten recombinant proteins have been produced in the milk of either goats, sheeps, rabbits or pigs [19]. Furthermore, several functional heterologous proteins, including lysostaphin [20], bovine casein [21] and hLF [22], have been produced via cattle mammary bioreactors. Although a cattle mammary bioreactor secreting functional rhLF at 2.8 mg/ml has been established [22], its low transgenic efficiency is attributable to the pronuclear microinjection technique used and the extensive waiting period required to establish the transgenic animals lines [23]. However, a combination of gene transfer in cultured somatic cells and somatic cell nuclear transfer techniques provide an attractive alternative to improve the transgenic efficiency. We thus employed this approach to produce large amounts of biologically active rhLF in the cattle mammary bioreactor. For high-level and stable expression of rhLF in transgenic animals, we had previously optimized the use of a construct carrying the entire hLF genomic sequence and obtained transgenic mice capable of producing rhLF at up to 8 mg/ml of milk [24]. Our results demonstrated that expression of rhLF by a bacterial artificial chromosome (BAC) containing the entire hLF genomic sequence is an effective means for the generation of transgenic animals capable of expressing high-levels of stable protein. However, because of its large size, a BAC is not easy to transfer into cells by conventional techniques. Microinjection is an effective technique for the introduction of large DNA fragments into cell nuclei but, to our knowledge, there have been no report**s** on the microinjection of a BAC into cultured cells to produce a livestock mammary bioreactor. Therefore, we pursued this goal by co-microinjecting a 150-kb BAC containing the entire hLF gene (including 90-kb and 30-kb 5′ and 3′ flanking regions) with a plasmid encoding a marker gene into bovine fetal fibroblast cells. With subsequent transgenic cloning, we obtained transgenic cattle that expressed a high-level of functional rhLF.

## Results

### Transfection of hLF BAC DNA

The hLF BAC was successfully integrated into bovine fibroblast cells by microinjection, with integration efficiencies of up to 15.79×10^−2^ percent (table 1). In our experiments, both electroporation and lipofection were unable to transfect the hLF BAC into cells (data not shown). It was also noted that the integration efficiency of plasmid pCEIN containing two marker genes, by microinjection was apparently higher than by either electroporation or lipofection (data not shown).

**Table 1:** Efficiency of co-transfection of hLF BAC and pCEIN by microinjection.

Manipulated cells	GFP and Neo^r^ positive colonies*		hLF BAC integration colonies†	
	Number	Efficiency(%)	Number	Efficiency(%)
2050	21	1.02	2	9.76×10^−2^
2300	17	0.74	2	8.70×10^−2^
1900	18	0.95	3	15.79×10^−2^

* After approximately 20 days, the positive colonies (expressing GFP and Neo^r^) were screened by G418 and confirmed by the expression of GFP.

† After the GFP and Neo^r^ positive colonies were selected and expanded, DNA extraction was performed using some cells from each colony, and the integrated cells were determined by PCR using primer P1, P2 and P3.

### Production of transgenic cattle

Of 623 reconstructed embryos, 280 developed to blastocysts. Among these, 98 randomly chosen blastocysts were transferred to 50 recipient cows (table 2). Ten cows became pregnant after embryo transfer, and five calves were born at full term (the others were spontaneously aborted). Finally, two calves, named 211 and Xiang, survived after weaning and both were apparently healthy. Three out of five calves died of gastrointestinal disease after birth. It is well established that some unknown mechanisms affect the development, growth and/or survival of cloned animals [25], [26]. Though neonatal losses are common in cloning and decrease the overall success rate, the surviving calves are almost always transgenic.

**Table 2:** Efficiency of steps in cloned process from oocytes to transgenic calves.

Step	Total No.	Percent*
Oocytes	844	–
Re-constructed embryos	623/844	73.8
Blastocysts	280/623	44.9
Transferred recipients	50/98†	51.0
Pregnant	10/50	20.0
Born alive	5/10	50.0
Alive after weaning	2/98	2.04

* Percent indicate the percentage of embryos obtained successfully each step.

† 96 blastocysts were transferred to 48 recipients with two blastocysts per recipient and 2 blastocysts were transferred to 2 recipients with one blastocyst per recipient.

### Identification of the rhLF transgene in cattle

**Figure 1:**
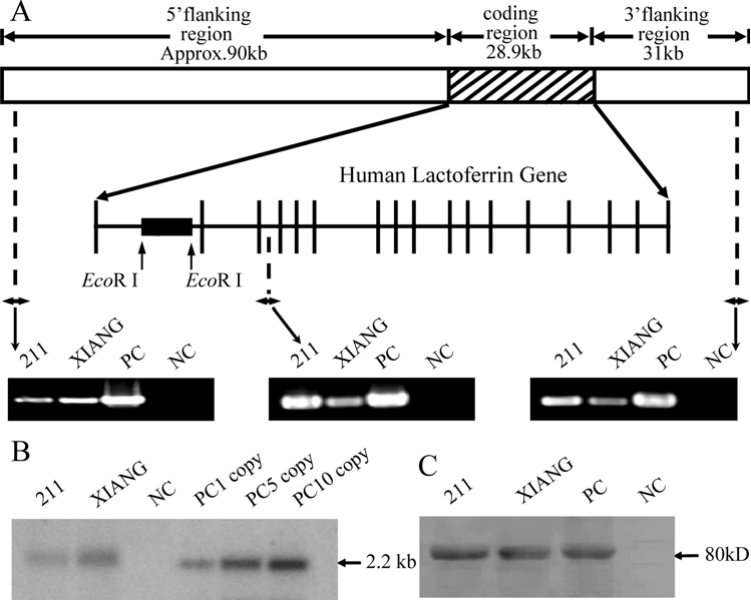
Results of determination of the hLF transgene. (A) Schematic representation of the transgene. A BAC containing the complete *hLF* genomic DNA (∼28.9-kb genomic sequence containing human *hLF* flanked by a 90-kb 5′ flanking and a 31-kb 3′ flanking region) were microinjected into bovine fetal fibroblasts. The black box showed the 2.2 kb EcoR I fragment used as probe in Southern blot. The positions of P1, P2 and P3 primers for PCR screening are indicated by arrows. (B) Southern analysis of DNA from transgenic calves. Genomic DNA (10 µg) was digested by *Eco*R I and hybridized by a ^32^P-labeled fragment. 211 and XIANG, transgenic calves; NC, non-transgenic calf; PC, positive controls with 1, 5 and 10 copies. (C) Western blot analysis of LF in milk. 211 and XIANG, transgenic milk; PC, human lactoferrin standard; NC, non-transgenic milk.

PCR results indicated that the 150-kb whole-genomic *hLF* sequence was introduced intact into the bovine genome (Figure 1A). Southern blotting confirmed the PCR results and showed that 211 and Xiang received one and two copies of the transgene, respectively (Figure 1B). Western blotting indicated that these two cows were likely to produce rhLF at high levels (Figure 1C). Analysis by radioimmunoassay (RIA) further demonstrated that rhLF was highly expressed in the transgenic milk, at concentrations of 2.5±0.2 g/l (211) and 3.4±0.4 g/l (Xiang). Fluorenscent *in situ* hybridization (FISH) analysis revealed that the BAC integrated into a single location in the genome of cattle (Figure S1).

### Composition analysis of transgenic milk

**Figure 2:**
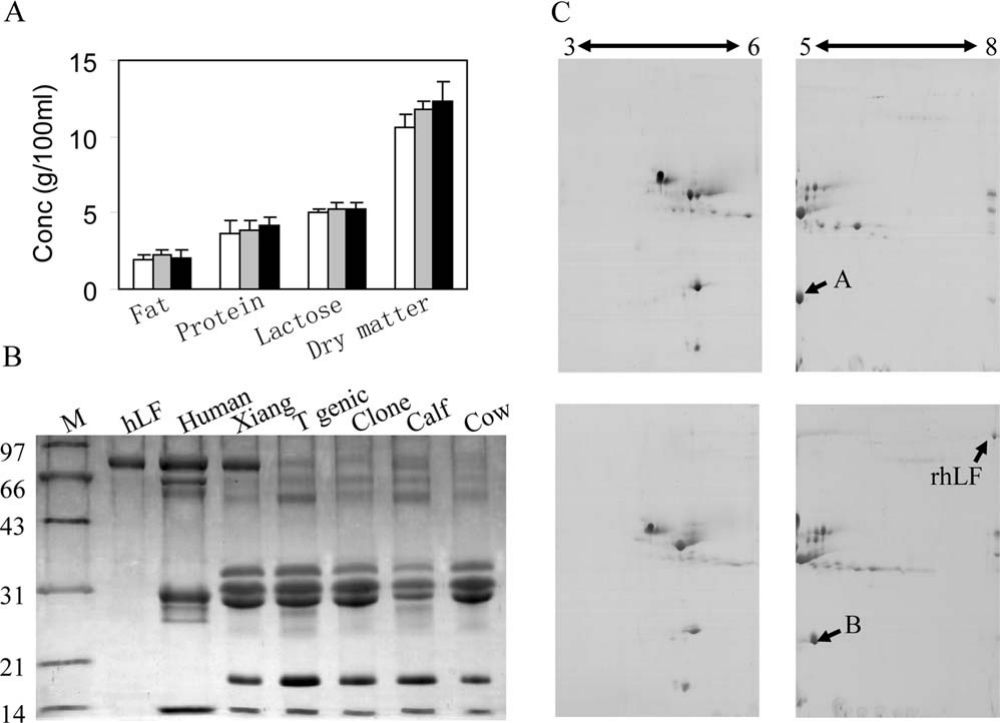
Composition analysis of transgenic milk. (A) Comparison of basic components of transgenic milk with those of conventional milk. Open bars, milk of 211; gray bars, milk of xiang; black bars, milk of non-transgenic cattle. (B) Analysis of proteins in whole milk via 15% SDS-PAGE. Whole milk (1.5 µl) of each group was loaded. M, protein marker; hLF, human lactoferrin standard; Human, human milk; Xiang, milk of hLF-transgenic calf xiang; T genic, milk of EGFP-NEO-transgenic calves; Clone, milk of cloned calves; calf, milk of non-transgenic calves; cow, milk of non-transgenic cows. (C) Global profiles of proteins expressed in non-transgenic milk (top) and transgenic milk (bottom) via two-dimensional gel electrophoresis. Total protein (25 µg) was loaded. The double-headed arrows indicate the range of pH. The protein spots labeled A and B correspond to β-lactoglobulin variant a and β-lactoglobulin variant b, respectively.

The gross composition of the transgenic milk, including fat, total protein, lactose and dry matter, was similar to that of non-transgenic milk (Figure 2A). Furthermore, the total protein profiles of whole milk from transgenic cattle, EGFP-NEO-transgenic cattle, cloned cattle, and non-transgenic cattle also were similar, with the exception of high-level expression of rhLF in transgenic milk (Figure 2B). Moreover, two-dimensional electrophoresis showed no obvious differences in the major milk proteins between transgenic milk and normal milk (Figure 2C). However protein B was detected in transgenic milk, whereas protein A was detected in normal milk. The two proteins had different isoelectric points. The results of peptide mass fingerprint showed that proteins A and B were β-lactoglobulin a and β-lactoglobulin b, respectively, which are constitutive components of normal milk (Figure S2C).

### Purification of rhLF from milk of transgenic cows

**Figure 3:**
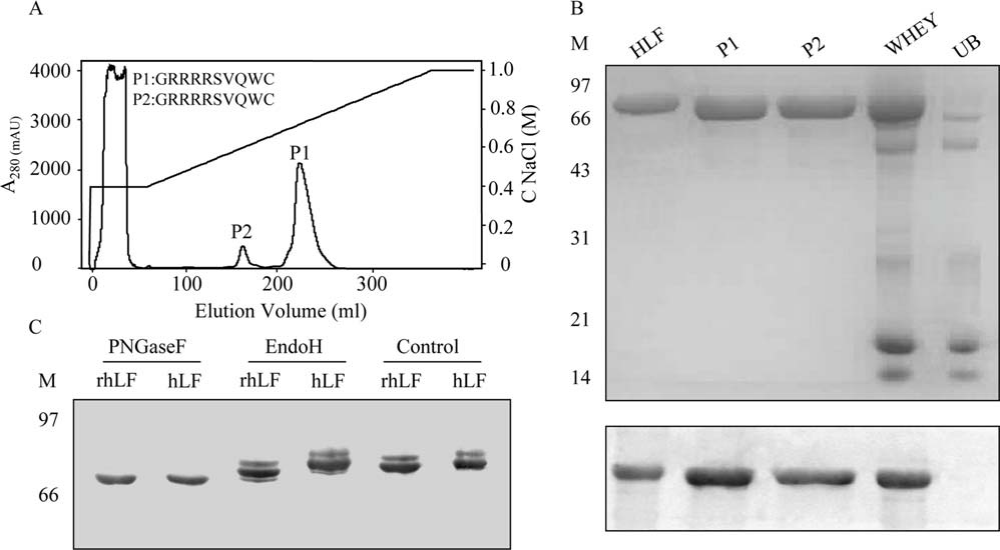
Purification of rhLF by cation exchange chromatography. (A) Profile of purification by liquid chromatography on a HiLoad 16/10 SP Sepharose HP column. Transgenic milk whey (15 ml) was loaded on the column. The N-terminal sequences of P1 and P2 are shown using the standard one-letter codes for amino acids. (B) Identification of rhLF by SDS-PAGE (15% gel, top) of SP Sepharose fractions and by western blotting (bottom). hLF (5 µg) was loaded as a standard. M, protein marker; hLF, human lactoferrin standard; P1 and P2, rhLF eluted from the column; WHEY, whey of transgenic milk; UB, protein fraction that was not bound to the column. (C) Western blot of rhLF (lanes 1, 3 and 5) and hLF (lanes 2, 4 and 6) treated with PNGaseF (lanes 1 and 2) or Endo H (lanes 3 and 4) or untreated (lanes 5 and 6). Samples were 5 µg in this experiment.

hLF contains many basic residues, and thus we used cation-exchange chromatography to purify rhLF. Two proteins, labeled P1 and P2, were eluted at 0.70 M and 0.60 M NaCl concentration, respectively (Figure 3A). Western blotting indicated that P1 and P2 both were rhLF (Figure 3B), which was further confirmed by peptide mass fingerprinting (Figure S2A) and N-terminal sequencing (Figure 3A). Furthermore, the efficiency of rhLF recovery from whole milk was 76%, as determined by ELISA (data not shown).

### Biochemical properties of rhLF

SDS-PAGE analysis of purified rhLF indicated that its apparent mass was slightly lower than that of hLF (Figure 3B). However, there was no difference in M_r_ after treatment with PNGase F (Figure 3C). Still, some differences were detected when the recombinant protein was treated with Endo H (Figure 3C). These differences may likely be caused by small variations in glycosylation patterns between rhLF and hLF; likely attributable to the slight difference in M_r_. The M_r_ of glycosylated rhLF obtained by MALDI-TOF was 79,494±20 Da (Figure S2B). Hence, the M_r_ of glycans of rhLF was estimated to be about 3 kDa by comparing the M_r_ of rhLF (the theoretical value of unglycosylated hLF is 76,320 Da).

### Susceptibility of rhLF to proteolysis

**Figure 4:**
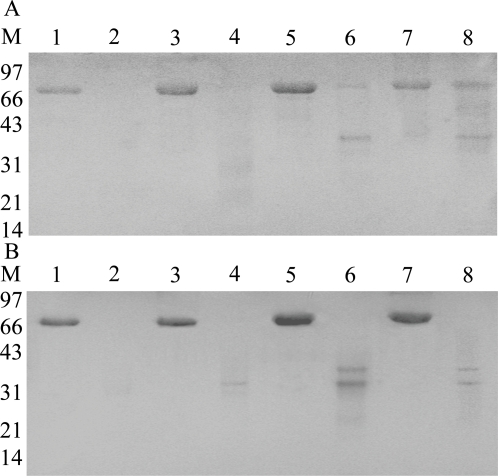
Proteolytic susceptibility of rhLF. (A) Western blotting results for rhLF (lanes 1, 2, 5 and 6) and hLF (lanes 3, 4, 7 and 8) treated with trypsin (lanes 2, 4, 6 and 8) or PNGaseF (lanes 1, 2, 3 and 4). (B) Western blotting results for rhLF (lanes 1, 2, 5 and 6) and hLF (lanes 3, 4, 7 and 8) treated with pepsin (lanes 2, 4, 6 and 8) or PNGaseF (lanes 1, 2, 3 and 4).

hLF is relatively resistant to degradation by trypsin, and this resistance to trypsin proteolysis is dependent on the extent/type of glycosylation on hLF [27]. Thus, we studied the susceptibility of glycosylated and unglycosylated forms rhLF to proteolysis by trypsin. Both unglycosylated rhLF and hLF were completely digested after treatment of 15 min (Figure 4A). We also studied susceptibility of rhLF to pepsin; both unglycosylated and glycosylated rhLF and hLF were completely proteolyzed after treatment of 1 h (Figure 4B).

### Iron binding and releasing properties of rhLF

**Figure 5:**
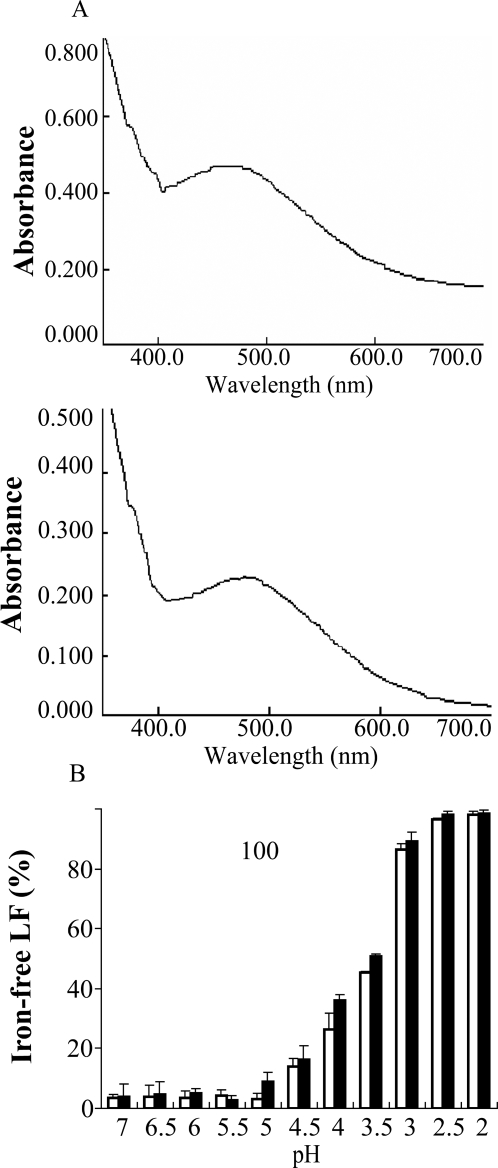
Iron binding and releasing properties of rhLF. (A) Determination of iron binding of rhLF (top) and hLF (bottom). (B) Profiles of iron release by rhLF (open bars) and hLF (black bars) as a function of pH.

One mole LF can bind two moles of metal ions accompanied by two moles of carbonate [1]. The absorbance spectrum at 465 nm of saturated LF is clearly altered from that of unliganded LF, indicating that LF binds to iron [13]. We analyzed the iron binding properties of rhLF and compared them with native hLF. The absorption peak of unliganded rhLF at 465 nm was clearly shifted after incubation with FeNTA solution for 1 hour (Figure 5A), suggesting that rhLF could bind iron. Iron can be released from the iron-saturated LF in acidic conditions [1]. We, therefore, studied the iron releasing properties of rhLF by incubating iron-liganded rhLF in solutions of varying pH. Iron release from rhLF was similar to that of hLF, and the release began to occur at ∼pH 4.5 and was complete at ∼pH 2.0 (Figure 5B). Iron-free LF had greater electrophoretic mobility on SDS-PAGE compared with iron-bound LF, apparently due to changes in tertiary structure (data not shown).

### Antibacterial effect of rhLF in vitro

**Figure 6:**
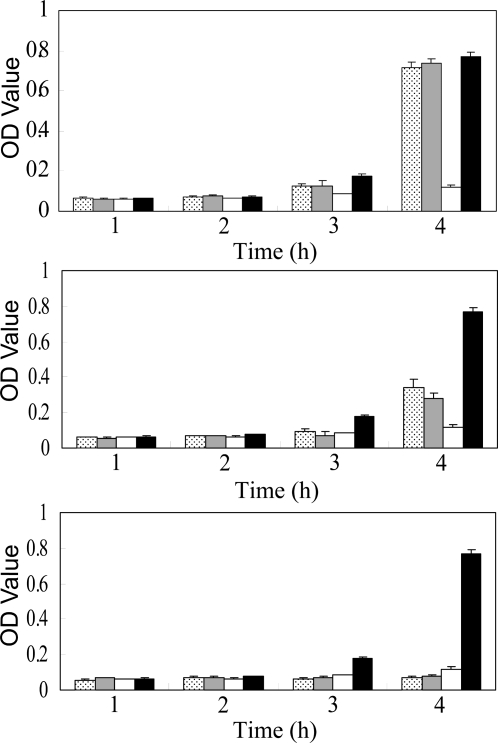
Antibacterial effects of rhLF and hLF. Antibacterial activity of rhLF at 0.5 mg/ml (top), 2 mg/ml (middle) and 5 mg/ml (bottom) on *E. coli* growth in liquid culture medium. Dotted bars, rhLF; gray bars, hLF; open bars, positive control (2 µg/ml ampicillin); black bars, negative control (nothing added). The experiment for each group was repeated at least three times, and the results represent means±s.d.

The ability of hLF to suppress bacterial proliferation is one of its most important properties; and thus, we tested rhLF for its effectiveness at inhibiting *E. coli* growth (Figure 6). The presence of 5 mg/ml rhLF significantly slowed *E. coli* proliferation compared to untreated controls. The suppressive effect of rhLF on bacterial growth was similar to that of hLF, and when 2 mg/ml of rhLF or hLF were used, *E. coli* proliferation was partially inhibited at the 4-hour time point.

## Discussion

To our knowledge, this is the first study in which a BAC containing the hLF gene has been expressed in the cattle mammary bioreactor. It is well established that additional regulatory elements may improve the expression of an exogenous gene, and BACs generally contain all the regulatory elements necessary for gene expression [28]. Therefore, the use of a BAC to carry a transgene may be the best alternative for producing stable recombinant proteins at a high and sustained level. In addition, the use of a BAC probably diminishes the “position effect”— caused by the chromosomal insertion site of an exogenous gene. Hence, we chose a 150-kb hLF BAC to carry the hLF transgene into bovine fetal fibroblasts to generate transgenic cattle. Our results suggest that the cattle mammary bioreactor is an efficient means for production of bioactive rhLF on a large scale.

Because of their large size, however, BACs are difficult to transfer into fibroblast cells by conventional techniques. In this study, 150 kb hLF BAC was not integrated into bovine fibroblast cells by electroporation or lipofection which were proved to be feasible for transfection of large DNA in other's studies[29]–[31]. Fortunately, it had already been reported that the microinjection technique is approximately one thousand times more efficient in producing gene-expressing cells than the transfection technique [32]. Furthermore, microinjection has been proven to be an effective approach for the transfection of plasmids containing exogenous gene into cells. However, there has been no reports on microinjection of large DNA fragment, such as BAC, YAC or PAC, into livestock cells. Zhang *et al.* reported transfection of YAC by polyethylene glycol-mediated spheroplast fusion [33]. Unfortunately, the average efficiency of fusion was approximately 36 per 2×10^6^ fibroblasts. Although many transgenic animals containing large-size DNA, have been obtained in the past decade, most have been performed in the mouse. Furthermore, gene transfer was primarily performed by pronuclear microinjection of large-size DNA, lipofection into ES cell or cell fusion with ES cell [28], [34], but all above gene transfer methods are notorious for low transfection efficiencies. We demonstrate, for the first time, the successful use of microinjection of bovine fibroblasts with a mixture of hLF BAC and a reporter gene to generate transgenic cattle. Moreover, the integration efficiency of BAC microinjection is notably higher than that of electroporation and lipofection. This suggests that microinjection provides an effective method for transfer of large-size DNA into cultured cells.

Although there is neither an official authorization or restrictive policy in China, the neomycin resistance gene still is extensively applied in transgenic research as a marker gene. In consideration of health concerns regarding both transgenic animals and humans, we investigated the expression of reporter genes in the tissue of the calves that died post-natal. Interestingly, the expression of the neomycin resistance gene and EGFP gene used as reporter genes in this study, were not detected in the heart, liver, spleen, lung, kidney, skin or muscle of the transgenic cattle as detected by radioimmunoassay (data not shown).

Although van Berkel *et al.* [22] reported the production of hLF transgenic cattle at expression levels of up to 2.8 mg/ml, the transgenic efficiency of 1.5% (2/129) was very low using pronuclear injection [23]. To overcome the experimental limitations of inefficient transgene integration that have long plagued researchers in the past, we combined the transgenic technique of microinjection and somatic cell nuclear transfer. The survived calves were all transgenic. We have, thus, resolved the problem of low transgenic efficiency by introducing the transgene into cultured cells followed by subsequent somatic cell nuclear transfer.

Moreover, animal usage was greatly economized in this study. A total of 844 oocytes were used and the efficiency of blastocyte formation was 45% (280/623), whereas the number of oocytes used in Krimpenfort's study was 2470 and the efficiency of blastocyte formation was 11% (129/1154) [23]. In our study, this is a 66% reduction in total oocytes required and double the blastocysts formed, compared to Krimpenfort. Furthermore, compared to pronuclear injection, our method is much more economical for generating transgenic cattle. Because we ensure that the reconstructed embryos are 100% transgenic before implantation, fewer recipients are required. To get two transgenic cows: 50 recipients were used in our study whereas 99 recipients were used in Krimpenfort's study, and 147 recipients were used in Eyestone's study [35]. Our study used 50% and 66% less recipients respectively.

As emerging biotechnological tools, the advance of cloning and transgene technique**s** provides the basis for an exciting future for large-scale production of nutrients and pharmaceutical proteins using the livestock mammary bioreactor. Moreover, previous studies suggest that the composition of milk and meat products and the general health of cloned animals are similar to those of non-transgenic animals [36]–[38]. Indeed, our comparison of the whole milk samples of cloned transgenic and natural cattle demonstrated that the composition of milk from cloned transgenic cows was not affected by transgene expression or the cloning process utilized in this study. As such, we anticipate future work will likely confirm this positive assessment of the quality and safety of transgenic milk.

Many efficient methods, such as ion-exchange chromatography [39], batch extraction [40] or reversed phase chromatography [41], have been used successfully to purify LF from milk. However, the greater the number of steps in the purification, the more product loss becomes an issue. Therefore, we used a single step of cation exchange chromatography to obtain highly purified LF. Our results revealed that p1 and p2, eluted at different NaCl concentration**s**, were identified to be rhLF by Western, N-terminal sequencing and MS analysis. We speculate that a low molecular weight whey component of negative charge interacts with rhLF in milk, thereby decreasing the net positive charge of rhLF [39]. Therefore, some of the rhLF was eluted at relatively lower NaCl concentration.

Comparison of rhLF and hLF on SDS-PAGE revealed a slight difference in relative M_r_ of ∼2 kDa, probably attributable to the differences in glycosylation because there was no M_r_ difference after deglycosylation. Our previous study revealed that rhLF expressed in murine milk had three types of glycosylation, at ratio of 2∶3∶5 [42]. The type of glycosylation that occurs on a recombinant protein is basically governed by the host species and the site of expression [43], and thus the actual molecular mass of a recombinant glycoprotein may vary from host to host [44]–[46].

Van Berkel *et al.* [27] showed that glycans play important roles in the proteolytic resistance of recombinant proteins. Moreover, intact hLF has been detected in feces of breast-fed infants [46], suggesting that hLF is not completely digested in the gastrointestinal tract. Here, we verified that rhLF, similar to native hLF, was to some extent resistant to proteolysis by trypsin. Unglycosylated rhLF and hLF were rapidly digested compared with their glycosylated counterparts. However, the recombinant protein was likely more susceptible than the native protein; Harri *et al.* [47] reported that the greater susceptibility of rhLF to proteolysis is mainly attributable to the mutations Ile^130^-Thr and Gly^404^-Cys rather than glycosylation of hLF. However, a bovine LF variant, bLF A — glycosylated at Asn^281^ — is more resistant to tryptic proteolysis than bLF B, which is unglycosylated at Asn^281^. Therefore, glycosylation of LF probably plays an important role in trypsin/proteolytic resistance.

hLF is thought to play a significant role in the transport and absorption of iron *in vivo* [48]–[50] in addition to antibacterial activities [51]. Therefore, we investigated the iron binding and releasing properties of rhLF to understand whether the recombinant protein, similar to native protein, chelated iron. In order to be used as a therapeutic agent, rhLF and hLF would need to have similar bioactivities, such as bacteriostasis and iron absorption, in newborns. Our results indicate that rhLF at 5 mg/ml can suppress bacterial proliferation, whereas at <2 mg/ml it does not have adequate bactericidal activity. Recently, Hyvonen et al. [52] reported that transgenic cows expressing rhLF at 2.9 mg/ml were not protected from experimental *E. coli* intra-mammary infection. Therefore, the effect of rhLF on bacteria mainly depends on its biologically relevant concentration. Regardless, rhLF indeed has the potential for use as a bactericide *in vitro*.

In conclusion, we report here, for the first time, the co-microinjection with a 150-kb hLF BAC and a marker gene into donor cells followed by somatic cloning is very efficient in producing cattle mammary bioreactor, and rhLF was expressed in the milk reaching a level of 3.4 g/l. Moreover, the biochemical properties and bioactivities of rhLF were similar to that of natural hLF. All of the above suggest that the novel procedure of transgenic cloning developed in this study is convenient and efficient in generating large animal mammary bioreactors, capable of great potential for the production of functional heterologous proteins on a large scale.

## Materials and Methods

### Preparation of the hLF gene

BAC clones containing the entire hLF genomic sequence (Genbank accession number: U95626) were obtained by screening a human BAC library (Genome Systems Inc.). A linearized 150-kb entire hLF genomic sequence was separated from the BAC clones (after digested with Not I) by pulse-field-gel-electrophoresis with CHEF mapper III (Bio-Rad, Hercules, CA) and recovered by electroelution prior to terminal sequencing for confirmation of intactness. Procedural details are presented in our previous work [24].

### Co-microinjection of hLF BAC and a marker gene into bovine fetal fibroblasts

Tissue biopsies were obtained from the skin of a day 142 bovine fetus, and fibroblasts from passage 3 to passage 5 were used to perform microinjection. The hLF BAC DNA (2.5 ng/µl) in TE buffer (10 mM NaCl, 10 mM Tris-HCl, 1 mM EDTA, pH 8.0) was mixed with pCMV-EGFP-IRES-NEO (pCEIN)(Clontech Inc., Palo Alto, CA) in a molar ratio of 1∶3, and this mixture was microinjected into bovine fetal fibroblast cells. After incubation and screening for 14 days at 600 µg/ml Geneticin (G418, Life Technologies, Carlsbad, CA), the resulting cells were collected and passaged twice in 300 µg/ml G418. PCR was performed to further screen the positive cells using three pairs of primers covering the complete *hLF* gene: FP1 (5′ TGCTTTGTTTGTATTGAGGGTC 3′) and RP1 (5′ CCAGGAACAAACTTACGGAG 3′, FP2 (5′ GATGCTGTGACCCTTGATGG 3′) and RP2 (5′ CATTCCATCCAGCGGTCC 3′), and FP3 (5′ TTCCTTCCACCACTGTTGAG 3′) and RP3 (5′ CAAATACCTCTGCCGCTGTT 3′). P1, P2 and P3 were designed to amplify the 5′ flanking region, coding sequence of hLF, and 3′ flanking region, respectively.

### Electroporation and lipofection

The hLF BAC DNA and pCEIN in TE buffer was co-introduced into bovine fetal fibroblast cells by electroporation. The electroporation procedures have been described elsewhere [53] and was modified in this study. Briefly 5×10^6^ cells in 400 ml Hepes-buffered saline were mixed with a final concentration of 2.5 ng/µl hLF BAC DNA and a final concentration of 0.5 ng/µl pCEIN and electroporated using a DC pulse of 1.2 kV/cm for 1 ms. Twenty-four hours later, G418 was added to select cells according to the method above. The lipofection was performed according to instruction of the Lipofectamine™ 2000 kit (invitrogen, Carlsbad, CA).

### Somatic cell nuclear transfer

Nuclear transfer was preformed as described [53]. Briefly, the nuclei of transgenic cells were transferred to enucleated oocytes to produce reconstructed embryos that were then electrically fused by a BTX 2001 Electro Cell Manipulator (BTX, San Diego, CA). The reconstructed embryos were activated with 10 µg/ml cycloheximide and 2.5 µg/ml cytochalasin-D in CR1aa medium [54]. Day-7 blastocysts were transferred to synchronous recipient cows with two embryos per recipient. The gestation of recipients were examined on day 60, 90, and 240. After birth of the calves, transgenics were identified by PCR and a copy number was assessed by Southern blot analysis.

### Composition analysis of transgenic milk

The intramuscular injection of medroxyprogesterone acetate (25 mg/kg/day) and estradiol benzoate (7.5 mg/kg/day) to cows at the age of eight months for seven days was carried out to induce lactation. Milk was collected for 14 days from the day one of of lactation. The composition analysis of whole-milk samples was performed on a MilkoScan 4000 (Foss, Hilleröd, Denmark). Western blotting was carried out with a rabbit polyclonal antibody against hLF (Biodesign, Saco, ME) and horseradish peroxidase–conjugated goat anti-rabbit IgG (Biodesign, Saco, ME).

### Two-dimensional electrophoresis

The concentration of total milk protein was measured with a Bradford Protein Quantity Assay Kit (Biyuntian, Beijing, China). For the first dimension of gel electrophoresis, defatted milk was subjected to a linear, immobilized pH gradient on 11-cm-long dry strips of pH 3–6 or pH 5–8 (Bio-Rad, Hercules, CA). The second dimension of gel electrophoresis was performed by 15% SDS-PAGE followed by staining with mass spectrometry–compatible methods [55]. The digitalized gel images were analyzed by Image Master Platinum version 6 software (GE Healthcare, Uppsala, Sweden).

### Purification of rhLF from transgenic milk

The rhLF was purified on an ÄKTA purifier 10 equipped with a HiLoad 16/10 SP Sepharose HP column (GE Healthcare, Uppsala, Sweden). Briefly, NaCl (final concentration of 0.4 M) was added to the transgenic milk prior to removal of milk fat and casein from the whey by centrifugation at 23,000×*g* for 60 min. Then the milk samples were diluted 5-fold in 20 mM sodium phosphate, 0.4 M NaCl, pH 7.5, filtered with a 0.22 µm filter and applied to the column. Bound proteins were eluted with a linear salt gradient of 0.4–1 M NaCl in 20 mM sodium phosphate, pH 7.5. The flow rate was at 3.0 ml/min, and the absorbance was measured at 280 nm. The absorbance peaks were integrated with UNICORN software (GE Healthcare, Uppsala, Sweden). The rhLF-containing fractions were desalted by ultracentrifugation using a 50-kDa cutoff ultracentrifuge tube (Centricon, Bedford, MA), and the retained solution was freeze-dried on Freezone 6 (Labconco, Kansas City, MO).

An ELISA kit (Merck, Darmstadt, Germany) was used to determine the efficiency of purification. The concentration of whey (before and after application to the column) and the pooled rhLF was determined by measuring absorbance at 420 nm on a Microplate Reader (Bio-Rad, Hercules, CA).

### Glycosylation analysis of rhLF

Deglycosylation of rhLF and native hLF with N-glycosidase F (PNGase F) and Endo H (New England Biolabs, Herts, UK) was performed as per the manufacturer's instructions. The rhLF solution (1 mg/ml) was treated with PNGase F (final concentration of 5 U/µl) and Endo H (final concentration of 5 U/µl) respectively, and successively boiled for 5 minutes in non-reducing SDS-PAGE sample buffer prior to analysis by 7.5% SDS-PAGE.

### Analysis of susceptibility to proteolysis

Proteolysis by trypsin (Sigma) was performed according to the manufacturer's instructions. Briefly, the trypsin (final concentration of 0.2 mg/ml) was added to a solution of rhLF (final concentration of 1 mg/ml) and incubated at 37°C for 15 min. The reaction was stopped by adding a 10-fold molar excess of Soybean trypsin inhibiter (Sigma).

Meanwhile, proteolysis by pepsin (Sigma) was carried out according to the manufacturer's instructions. The rhLF and native hLF were dissolved in distilled water at a concentration of 5 mg/ml with a final pH of ∼2.0. Pepsin was then added to each solution at a final concentration of 0.5 µg/ml and incubated at 37°C for 1 h. The reaction was terminated by adding 0.5 µg/ml (final concentration) pepstatin A (Sigma). The resulting peptides were subjected to 15% SDS-PAGE and transferred to a nitrocellulose membrane as described above.

### Iron saturation and desaturation of rhLF

The purified protein was saturated with freshly prepared FeNTA solution as described [27]. Briefly, the FeNTA solution (10 mM ferric nitrate, 8.5 mM nitrilotriacetic acid) was adjusted to pH 7.0 with solid NaHCO_3_. rhLF was added to the FeNTA solution to achieve a molar ratio of rhLF to iron of 1∶4, and the solution was incubated at 20°C for 1 h. The resulting iron-saturated rhLF was dialyzed against 0.15 M NaCl. Spectral analysis was performed from 260 to 700 nm on a Lincam spectrophotometer (Lincam, Cambridge, UK).

To measure the potential of rhLF to bind iron under different conditions, iron-saturated rhLF solutions (5 mg/ml) were dialyzed for 36 h at room temperature against the following buffers, each of which contained 0.15 M NaCl: 0.1 M HEPES (pH 7.0); 0.1 M MES (pH 6.5–5.5); 0.1 M sodium acetate (pH 5.0–3.5); 0.1 M glycine/HCl (pH 3.0–2.0). Absorbency at 280 nm and 465 nm was measured on a spectrophotometer. The concentration of iron-free rhLF was calculated as: (A_280_−1.4×(A_465_/0.058))/1.1 [18]. The proportion of iron-free LF was calculated as the ratio of iron-free rhLF to total rhLF.

### Analysis of rhLF antibacterial activity *in vitro*

*Escherichia coli* F107 was obtained from the Conservation Institute of Chinese Veterinary Microbacteria, Agricultural Ministry of China. Cells (10^5^ CFU/ml in broth agar medium) were incubated at 37°C with agitation (240 rpm/min) with rhLF solution (final concentration of 0.5 mg/ml, 2 mg/ml, or 5 mg/ml in broth agar medium). A negative control was prepared with distilled water in place of rhLF. The positive control was 2 µg/ml ampicillin solution in place of rhLF. Aliquots (100 µl) of samples taken at different times were measured at 600 nm to detect the growth of bacteria. The assays were carried out at least three times.

## Supporting Information

Figure S1 Localization of the rhLF transgene in chromosomes of the transgenic calves by fluorescence in situ hybridization. The arrows indicate the location of rhLF gene on chromosome 15 in 211 (A) and xiang (B).WT is untransgenic cattle.
(2.44 MB TIF)

Figure S2 Investigation of rhLF in transgenic milk with MS. A, The results of P1 (top) and P2 (bottom) analyzed by LCQ Deca xp^plus^ mass spectrometer. The red sequences indicate the peptides identified by mass spectrometer. B, Determination of M_r_ of rhLf with MALDI-TOF MS. The M_r_ of rhLF is 79,494 Dalton. C, Identification of different proteins, A (left) and B (right), in two dimensional electrophoresis with MALDI-TOF MS. Match sequences of A and B were beta-lactoglobulin variant a and beta-lactoglobulin variant b of cattle, Sequence coverage of beta-lactoglobulin variant a and beta-lactoglobulin variant b were 44% and 50% respectively.
(2.88 MB TIF)
