# Supplementary figures and images for: Cattle Mammary Bioreactor Generated by a Novel Procedure of Transgenic Cloning for Large-Scale Production of Functional Human Lactoferrin
Source: PLoS One. 2008 Oct 20;3(10):e3453. doi: 10.1371/journal.pone.0003453 (PMC2565487; doi:10.1371/journal.pone.0003453)

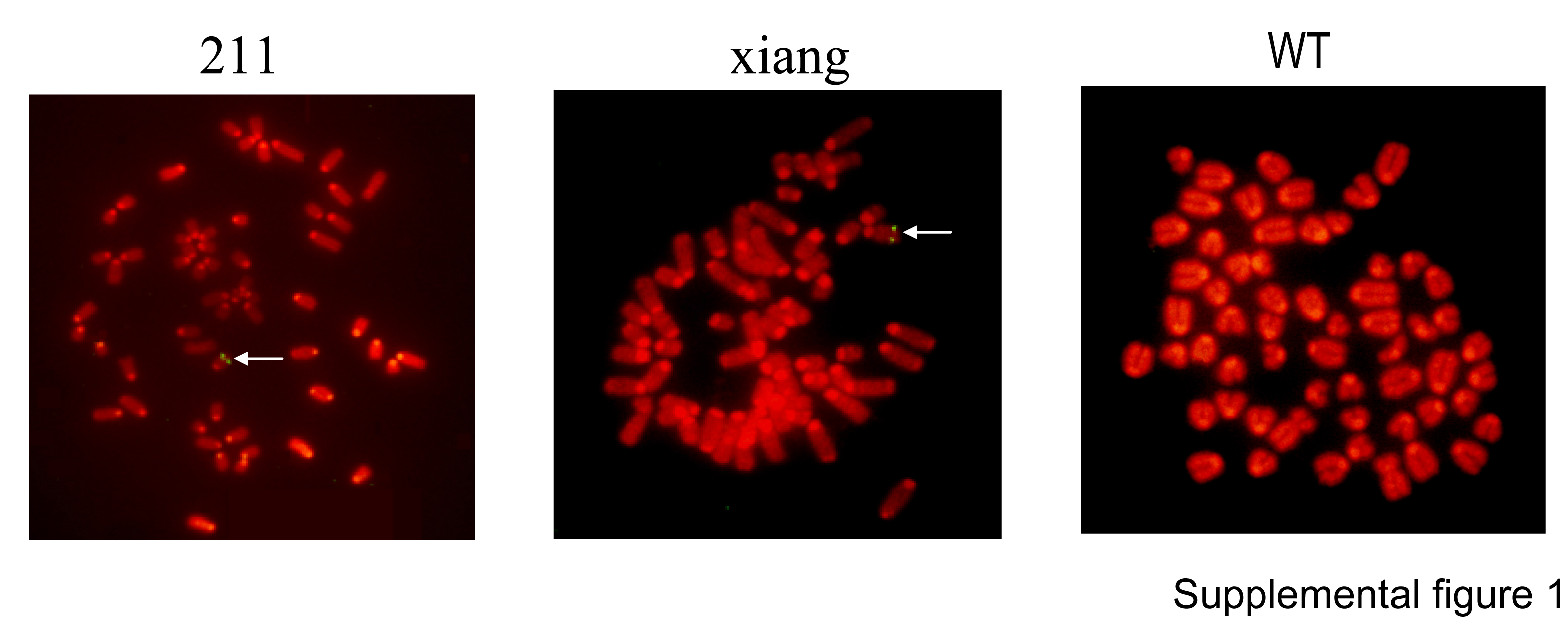

Supplement: Figure S1 — Localization of the rhLF transgene in chromosomes of the transgenic calves by fluorescence in situ hybridization. The arrows indicate the location of rhLF gene on chromosome 15 in 211 (A) and xiang (B).WT is untransgenic cattle. (2.44 MB TIF) [file pone.0003453.s001.tif]

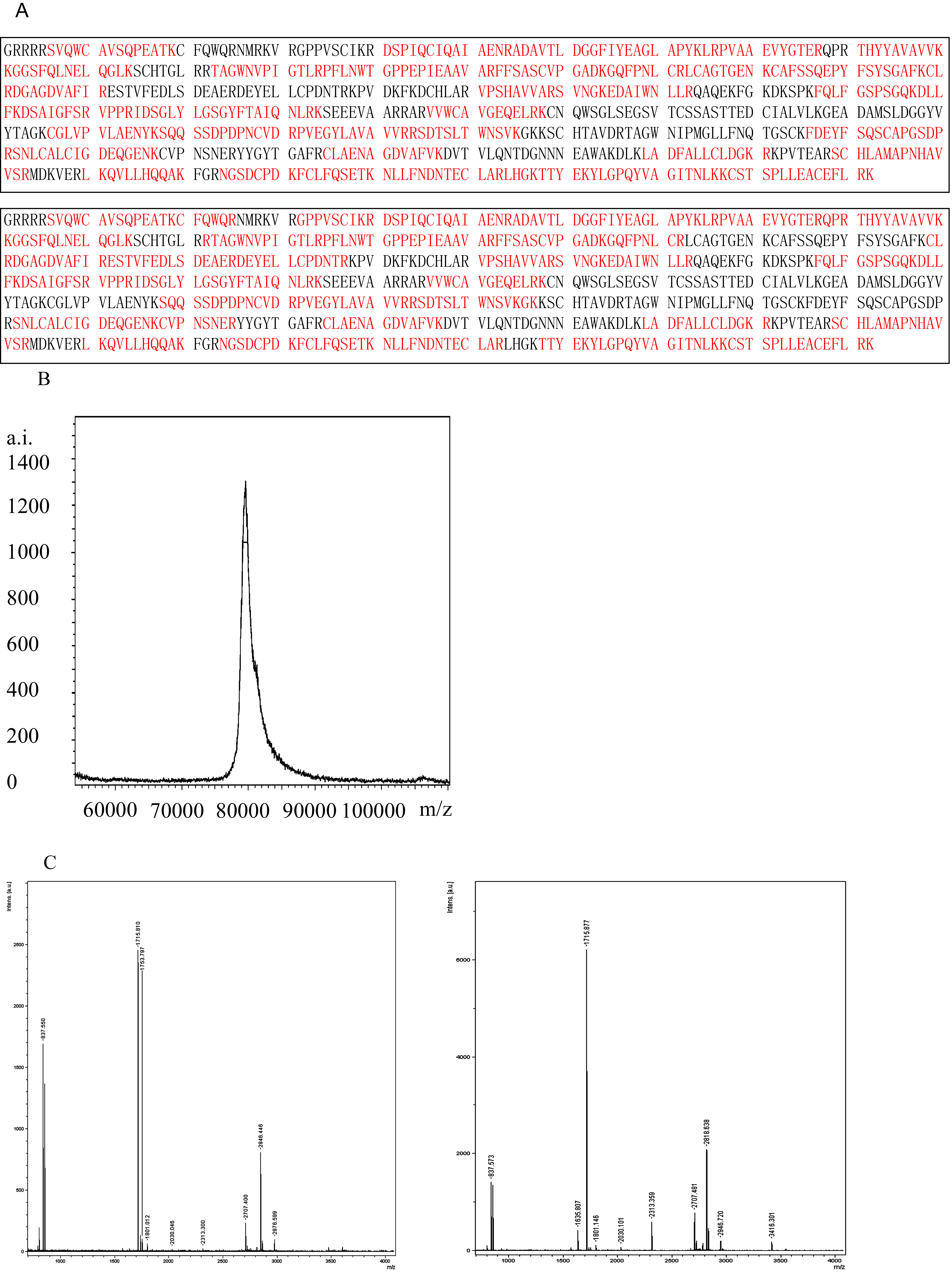

Supplement: Figure S2 — Investigation of rhLF in transgenic milk with MS. A, The results of P1 (top) and P2 (bottom) analyzed by LCQ Deca xpplus mass spectrometer. The red sequences indicate the peptides identified by mass spectrometer. B, Determination of Mr of rhLf with MALDI-TOF MS. The Mr of rhLF is 79,494 Dalton. C, Identification of different proteins, A (left) and B (right), in two dimensional electrophoresis with MALDI-TOF MS. Match sequences of A and B were beta-lactoglobulin variant a and beta-lactoglobulin variant b of cattle, Sequence coverage of beta-lactoglobulin variant a and beta-lactoglobulin variant b were 44% and 50% respectively. (2.88 MB TIF) [file pone.0003453.s002.tif]
